# Web alert: Fabrication with microbial spores

**DOI:** 10.1111/1751-7915.14345

**Published:** 2023-09-27

**Authors:** Lawrence P. Wackett

**Affiliations:** ^1^ Department of Biochemistry, Molecular Biology and Biophysics, BioTechnology Institute University of Minnesota St. Paul Minnesota USA

Printing spores

https://pubmed.ncbi.nlm.nih.gov/31792444/

This abstract links to a paper, a commentary, similar articles and papers citing this paper. The focus of the paper was on embedding spores within hydrogels.

Bacterial spores ecology and biotechnology

https://pubmed.ncbi.nlm.nih.gov/30798805/

This links to an abstract and provides additional links to related papers. The main paper is a review article that highlights applications of spores and calls for more work on the ecology of spores and their role in the environment.

Applications of *Bacillus* spores

https://journals.asm.org/doi/10.1128/aem.01096‐20

This review article highlights the applications of *Bacillus subtilis* spores. Topics include recycling enzymes, drug delivery and applications in materials science.

Bioengineered *Bacillus* spores

https://pubs.acs.org/doi/10.1021/acssynbio.0c00578

This study explored the engineering of the spore forming and releasing processes while using a minimum of genetic modifications.

Spore surface display

https://www.jmb.or.kr/journal/view.html?doi=10.4014/jmb.1807.06066

This paper reviews the most important aspects of spore surface display systems with a particular focus on its applications.

*Bacillus* journal club: Spores

https://www.linkedin.com/pulse/bioprinting‐bacillus‐subtilis‐spores‐daniel‐zeigler

This post discusses a paper describing *Bacillus* spore imprinting and offers additional ideas as to how that technology could be used.

Engineering spores

https://academiccommons.columbia.edu/doi/10.7916/53g9‐y771

This page links to a doctoral thesis that presents details on the engineering of spore processing to express a heterologous protein in the spore protective coat.

Mechanisms of spore germination

https://pubmed.ncbi.nlm.nih.gov/37125461/

This study examined *Bacillus* and *Clostridium* species spores. The major focus was on food sterilization.

Spore delivery systems

https://www.mdpi.com/2218‐273X/13/6/947

This review article discusses the potential applications for spore delivery mechanisms to make new mucosal surface vaccines.

Spore germination receptors

https://www.cell.com/trends/microbiology/fulltext/S0966‐842X(23)00165‐8

While spores are relatively dormant, they must have sensing mechanisms to know when they can acquire specific environmental nutrients that will allow them to germinate. This article discussed these mechanisms.

How spores come back to life

https://www.sciencedaily.com/releases/2023/04/230427173433.htm

This news article highlights a paper in the journal *Science* that reports on spore sensing mechanisms and germinations.

Fabrication of inhalable spores

https://www.sciencedirect.com/science/article/abs/pii/S0378517312002852

This paper does not use biological spores but takes inspiration from natural spore delivery into lungs to generate nanomaterials for drug delivery.

Artificial spores

https://www.nanowerk.com/spotlight/spotid=27711.php

This is a synopsis of a paper describing artificial spores. The procedure described is to coat living cells with various materials to make a more stable, long‐lasting biological material.

